# Noninvasive Young's modulus visualization of fibrosis progression and delineation of pancreatic ductal adenocarcinoma (PDAC) tumors using Harmonic Motion Elastography (HME) *in vivo*

**DOI:** 10.7150/thno.37965

**Published:** 2020-03-15

**Authors:** Alireza Nabavizadeh, Thomas Payen, Alina C. Iuga, Irina R. Sagalovskiy, Deborah Desrouilleres, Niloufar Saharkhiz, Carmine F. Palermo, Stephen A. Sastra, Paul E. Oberstein, Vilma Rosario, Michael D. Kluger, Beth A. Schrope, John A. Chabot, Kenneth P. Olive, Elisa E. Konofagou

**Affiliations:** 1 Department of Biomedical Engineering, Columbia University Irving Medical Center, New York, NY 10032.; 2 Division of Hematology & Oncology, Department of Medicine, Columbia University Irving Medical Center, New York, NY 10032.; 3 Herbert Irving Comprehensive Cancer Center, Columbia University Irving Medical Center, New York, NY 10032.; 4 Division of Digestive and Liver Diseases, Department of Medicine, Columbia University Irving Medical Center, New York, NY 10032.; 5 Department of Pathology & Cell Biology, Columbia University Irving Medical Center, New York, NY 10032.; 6 Division of GI/Endocrine Surgery, Department of Surgery, Columbia University Irving Medical Center, New York, NY 10032.; 7 Laura and Isaac Perlmutter Cancer Center, New York University, New York, NY.

**Keywords:** PDAC: Pancreatic Ductal Adenocarcinoma, HMI: Harmonic Motion Imaging, HME: Harmonic Motion Elastography, PSR: Picrosirius Red, YM: Young's Modulus.

## Abstract

**Background and aims**: Poor specificity and predictive values of current cross-sectional radiological imaging methods in evaluation of pancreatic adenocarcinoma (PDAC) limit the clinical capability to accurately stage the tumor pre-operatively and provide optimal surgical treatment and improve patient outcomes.

**Methods**: In this study, we applied Harmonic Motion Elastography (HME), a quantitative ultrasound-based imaging method to calculate Young's modulus (YM) in PDAC mouse models (n = 30) and human pancreatic resection specimens of PDAC (n=32). We compared the YM to the collagen assessment by Picrosirius red (PSR) stain on corresponding histologic sections.

**Results**: HME is capable of differentiating between different levels of fibrosis in transgenic mice. In mice without pancreatic fibrosis, the measured YM was 4.2 ± 1.3 kPa, in fibrotic murine pancreata, YM was 5.5 ± 2.0 kPa and in murine PDAC tumors, YM was 11.3 ± 1.7 kPa. The corresponding PSR values were 2.0 ± 0.8 %, 9.8 ± 3.4 %, and 13.2 ± 1.2%, respectively. In addition, three regions within each human surgical PDAC specimen were assessed: tumor, which had both the highest Young's modulus (YM > 40 kPa) and collagen density (PSR > 40 %); non-neoplastic adjacent pancreas, which had the lowest Young's modulus (YM < 15 kPa) and collagen density (PSR < 10%) and a transitional peri-lesional region between the tumor and non-neoplastic pancreas with an intermediate value of measured Young's modulus (15 kPa < YM < 40 kPa) and collagen density (15% < PSR < 35 %).

**Conclusion**: In conclusion, a non-invasive, quantitative imaging tool for detecting, staging and delineating PDAC tumor margins based on the change in collagen density was developed.

## Introduction

Pancreatic ductal adenocarcinoma (PDAC) has the shortest survival time of any major cancer in the developed world 1, 2. In the United States, it will be the second leading cause of cancer-related deaths by 2030 3-8. This is in part a consequence of late diagnosis: initial symptoms are typically non-specific and generally arise after the tumor has progressed. Radiologically, it is challenging to accurately delineate PDAC tumor's margins. Current MRI and CT imaging scanners are not specific enough to accurately differentiate tumor from adjacent non-neoplastic fibrotic pancreas with chronic pancreatitis. The limitations of current technologies for assessing the extent of the tumor make surgical decisions difficult 9-12. According to the international Study Group of Pancreatic Surgery, imaging specificity ranges from 67 to 91% due to inadequate differentiation between soft tissue scarring and true cancerous invasion 9. This is supported by the Society of Abdominal Radiology and American Pancreas association consensus statement, which described a high positive predictive value of CT to determine non-resectability but only a 45-79 % value for predicting resectability 10.

A prominent feature of PDAC is the presence of a profuse desmoplastic stroma that is associated with altered mechanical properties of the tissue. As a result, PDAC is one of the stiffest of all human tumors, an attribute that has been associated with highly increased solid stress and interstitial fluid pressure (IFP) 6, 13, 14. The high mechanical pressure of PDAC impedes tissue perfusion, collapses blood vessels, and interferes with drug delivery 8, 15, contributing to their resistance to radiation, cytotoxic, and molecularly targeted therapies 1-8, 16-19.

Collagen fibers are main tissue components with a crucial role in maintaining structural integrity in normal tissue, while playing a vital role in pathological processes such as inflammation, which often culminates in fibrosis or scarring 20. Similarly, collagen is the main component of extracellular matrix (ECM) in PDAC. The desmoplastic reaction in PDAC is mostly managed by collagen I 21-24. Disruption of the basement membrane in PDAC leads to elevated amounts of interstitial collagen, with protumorigenic effects 21, 23). The high level of collagen I deposition has been related to decrease in survival 25.

Pancreatic stromal fibrosis renders differentiation between chronic pancreatitis (CP) and neoplasia difficult. CP is considered a risk factor for PDAC and differentiating between these two processes is difficult. Radiologically, it is challenging to accurately distinguish tumors from chronic pancreatitis (CP). Intraoperative delineation of tumor versus adjacent fibrotic, non-neoplastic pancreas is difficult as well. In addition, the histological differential diagnosis between chronic pancreatitis and PDAC can be notoriously difficult 17, 21.

In order to investigate how to differentiate between PDAC and CP, we need to use well characterized animal models to control for the degree of fibrosis in tumor and non-neoplastic setting. Genetically engineered PDAC mouse models, which are physiologically and molecularly similar to their human counterpart, are available 8. Two recent studies using transgenic mice to study PDAC and CP showed that there is a correlation between tumor stiffness and its fibrosis stage 6, 13. However, the method used for stiffness estimation is invasive and applied only on small samples of pancreas in their mouse study.

Generally, to evaluate the mechanical properties of such tumors, in particular their stiffness, rheometry 26 and Atomic Force Microscopy 6 are the most common methods used. However, their application requires biopsy and is limited to small biopsy samples of a solid tumor that may show a significant degree of heterogeneity. In addition, it should be noted that the mechanical properties of the tumor in its original environment might not be the same as those of the excised one. Thus, a noninvasive method that can be used *in vivo*, with the ability to generate a 2D stiffness map is needed to provide important information on the structural tissue alterations in tumor progression 27.

Elastography is a modality for measuring tissue elasticity. Typically, this modality is combined with anatomical imaging such as ultrasound to provide an insight about the local properties of the medium 28, 29. In regard to excitation source, elastography techniques can be categorized in two main subgroups: dynamic elastography and quasi-static strain elastography. The former one is based on dynamic force stimulus and in the latter one, compressive force is applied 28, 29. Shear wave elastography is a dynamic elastography method in which either an external or internal excitation source such as acoustic radiation force can be applied 28, 29. Several techniques have been introduced based on the acoustic radiation force, including shear wave elasticity imaging (SWEI) methods 30, supersonic shear wave imaging (SSI) methods 31, acoustic radiation force impulse (ARFI) 32, shear wave dispersion ultrasonic vibrometry (SDUV) 33, comb-push ultra-sound shear elastography (CUSE) 34, harmonic motion imaging (HMI) 35 and harmonic motion elastography (HME) 36.

Shear wave elastography has had recent success in clinical translation, especially in liver fibrosis and tumor characterization 28. Although there have been several studies using shear wave methods to assess the stiffness of PDAC tumors and non-neoplastic tissue 37- 41, none of them distinctly report the Young's modulus values for the perilesional regions, the region located between the tumor and non-neoplastic region non-invasively.

Payen *et al.* 42, 43 described an *in vivo* application of Harmonic Motion Imaging (HMI) to differentiate the various levels of fibrosis in transgenic mice models. In this work, a quantitative methodology called Harmonic Motion Elastography (HME) confirmed the previous HMI-based qualitative observation. In addition, its local Young's modulus value was validated against histochemistry. Thus, the overall goal of this study is to quantitatively assess the mechanics of fibrosis progression in PDAC tumors as stiffening increases during that process 42, 43.

The aim of this study is to evaluate the HME performance in measuring stiffness and delineating the boundaries of PDAC tumor by estimating the Young' modulus (YM), mechanical stiffness standard unit (R^2^ = 0.95) 30, of pancreatic tumors with various degrees of fibrosis in transgenic mice *in vivo* and human surgical pancreatic specimens. The HME performance in transgenic mice with various degrees of pancreatic fibrosis ranging from wild-type, chronic pancreatitis and PDAC is described first, followed by human surgical pancreatic specimens, including chemotherapy and radiotherapy treated and untreated tumors. Finally, the capability of HME method to differentiate between cancerous and non-cancerous regions within each specimen was evaluated. In order to validate the HME results, Picrosirius red staining is used to visualize and quantify the fibrosis in all the murine pancreata and surgical human specimens, accordingly.

## Methods

### Harmonic Motion Elastography (HME)

**Figure 1:**
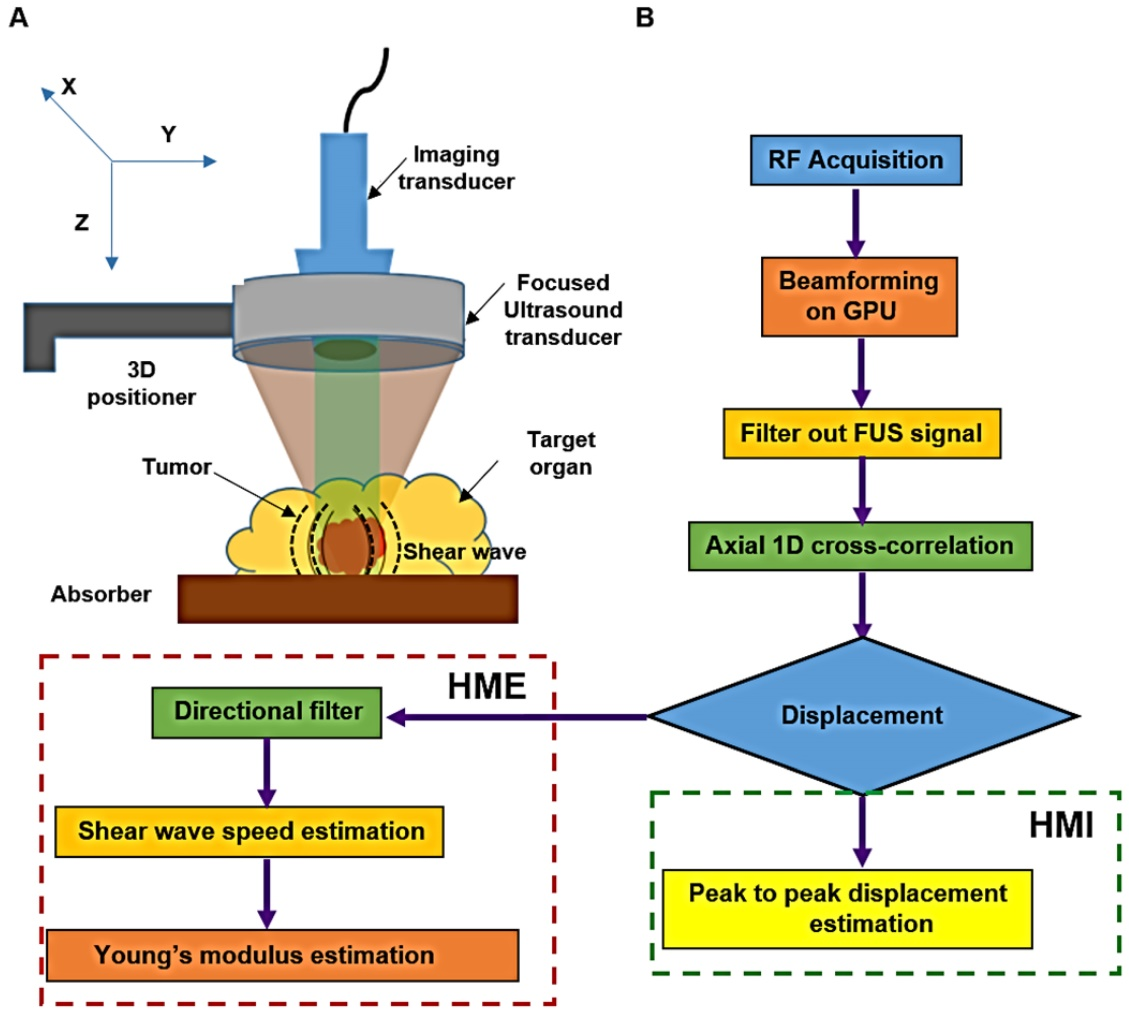
**A:** Harmonic Motion Elastography (HME) set up. **B:** Flowchart showing Harmonic Motion Elastography (HME) Vs. Harmonic Motion Imaging (HMI).

The HME setup, Figure 1, consists of a 93-element FUS (Focused Ultrasound) transducer (*f*c = 4.5 MHz, and *D* = 70 mm, Sonic Concepts Inc., Bothell WA, USA). For the imaging component we used a 64-element phased array imaging probe (fc = 2.5 MHz, P4-2, ATL/Philips, Bothell, WA, USA) to evaluate human pancreatic specimens and a 104-element diagnostic transducer (*f*c = 7.8 MHz, P12-5, ATL/Philips, Bothell, WA, USA) for the murine study 36. Each imaging transducer was confocally aligned with the HIFU transducer. The FUS transducer was derived by an AM sinusoidal signal generated by a dual-channel arbitrary waveform generator (AT33522A, Agilent Technologies Inc. Santa Clara, CA, USA) through a 50dB power amplifier (325LA, E&I, Rochester, NY, USA). In order to reduce the breathing motion artifact recorded during the *in vivo* mouse study, a respiration gating system (Biopac System, Santa Barbara, CA, USA) is used. The pressure sensor of this unit is connected to the MP150 Data Acquisition System. Its output is used to trigger the ultrasound Verasonic system (Vantage, Verasonics, Kirkland, WA, USA) to synchronize sonication and data acquisition 36, 42.

An amplitude-modulated wave form with an acoustic intensity of 1050 W/cm^2^ and frequency of force oscillation at 25 Hz, resulting in a 50 Hz displacement oscillation in the tissue, was applied for 0.6 s 42. An imaging probe at 1000 frames/s during the force application was used to record the data. The channel data acquired by the imaging transducer (Vantage, Verasonics, Kirkland, WA, USA) was used to generate the oscillatory deformation. To reconstruct each RF data frame from the channel data, the channel matrix was multiplied by the reconstruction sparse matrix. Its product matrix was multiplied by another sparse matrix for scan conversion. The entire beamforming process was performed by GPU (Graphical Processing Unit) in which the data recorded by the 64-element phased array on the surgical specimens, were up sampled at 80 MHz and the mice data recorded by the 104-element transducer were up sampled at 125 MHz 44. To estimate the axial displacement in a focal point, a 1D normalized cross correlation was applied on the reconstructed RF data 45. This procedure was similar in both HMI and HME methods. In the HMI method, the peak-to-peak of the resulted oscillatory displacement was calculated to assess the stiffness of the tissue 35. Thus, softer tissues undergo higher displacement compared to stiffer ones. However, in HME, the extracted shear wave from the oscillatory displacement is used to quantify the stiffness and generate the Young's modulus (YM, kPa) 2D map 36. In other words, the radiation force generates a complex wave field inside inhomogeneous tissue, rendering the shear wave speed estimation challenging. In order to extract the shear wave speed directionality we used directional filtering. This spatio-temporal filter 46, 47 works in the frequency domain and has the capability of selecting the portion of the wave along a specific direction, which culminates in disassembling the resulted complex wave field into its components that are propagating in different directions 34, 46-48. As we indicated previously, in the HME method, the directional filter is applied on the displacement data estimates to map the shear wave propagation (Fig.1). Then, by cross-correlating and measuring the delay that the shear wave incurs while traveling between two specific points in the same lateral direction at a fixed distance (six and eight wavelengths in mice and human specimens, respectively) the shear wave speed, 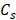 is measured. In other words, the ratio between the distance and the time delay that the shear wave takes to travel between these two points results in the shear wave speed measurement 34, 48.

Assuming that the soft tissues are locally incompressible, isotropic, and linearly elastic, the Young's modulus, 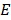 can be estimated from Eq.1 using the measured shear wave speed, 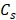.


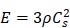
, (1)

where 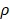 is the density assumed to be 1000 kg/m^3^ for all soft tissue. Due to the relatively larger size of the surgical specimens compared to the murine pancreata, we used raster scanning to cover the whole specimen. For each point, the 2D Young's modulus map is reconstructed first, then the resulting Young's modulus maps are concatenated according to their location of measurement to provide the final Young's modulus 2D map. However, to generate the 2D HMI map, as it is shown in Figure 1, the amplitude (peak to-peak) of displacement data is measured, showing that it is higher for softer tissues than stiffer ones, Figure 1B.

### *In vivo* Young's modulus assessment of transgenic mouse pancreata

The goal of this study was to evaluate the HME performance *in vivo*. Therefore, for this *in vivo* mice study, the protocol was reviewed and approved by the Institutional Animal Care and Use Committee (IACUC) of Columbia University. Within less than one hour, 1-2% isoflurane in oxygen was used to anesthetize the animal that was placed on a heating path in a supine position 36, 42. We located the pancreata with B-mode ultrasound imaging at 18.5 MHz (L22-14v, Verasonics). For spatial correlation between the HME system, B-mode and the high-frequency images we used a marker located above the mouse, in the water container 42. The entire pancreas was scanned for this study. Then the corresponding 2D Young's modulus maps were generated. We mapped the tail of the pancreas (TOP), the head of pancreas (HOP) or the body of pancreas (BOP), depending on where fibrosis or tumor were located. The measurement of fibrosis was also performed on corresponding regions after dissecting and slicing the pancreas.

### Mouse model characterization (chronic pancreatitis and PDAC models)

Forty-nine Young's modulus (YM) measurements in 30 mice in either head, body or tail of pancreas (HOP, BOP, TOP) depending on tumor location were performed. The control group included 18 YM measurements of 9 wild type BalbC mice for which both TOP and HOP pancreatic regions were assessed. This experimental group included also mice with chronic pancreatitis or KC (K-ras^LSL.G12D/+^; PdxCre^tg/+^) mice 8, 42, 43.There were a total of 26 YM measurements performed in 16 mice with chronic pancreatitis located in HOP and TOP. These mice can develop pancreatic adenocarcinoma when they are more than one-year-old. The mice are very susceptible to spontaneous inflammation with mild fibrosis and, at advanced age, this inflammation may lead to PDAC 43, 49, 50. Moreover, the inflammation effect can be drastically accentuated by administration of cerulein, a peptide analog of cholecystokinin. We injected cerulein (250 mg/kg daily for 5 days) in 8-20 week old, tumor-free KC mice to induce chronic pancreatitis 7. One week later, their whole pancreas was scanned using the HMI system. The mice were euthanized and necropsied immediately after scanning and pancreatic dissection was performed. After retrieval, the pancreata were immersed in cold 4% formaldehyde solution in phosphate-buffered saline (Affymetrix) at 4°C. Then, they were embedded in paraffin and cross-sectioned in 4 µm-thick sections. After deparaffinization, the samples were rehydrated and processed for routine staining with hematoxylin and eosin (H&E) using the ST Infinity H&E staining kit (Leica) 43. Further on, the Picrosirius red staining method was applied. A 0.1% Sirius Red solution dissolved in aqueous saturated picric acid was used in this staining protocol. First, the slides were incubated in this solution then washed with 100% ethanol. After that, they were dehydrated and mounted with Permount. After preparation, the freshly PSR stained slides were examined under polarizing light microscopy (Olympus BX41 TF) at a magnification of x 4. For all images, the halogen lamp intensity was set constant and an exposure time within each image type was selected and kept constant to help optimize the signal-to-noise ratio. CellSense acquisition platform (Olympus) was used to capture the images digitally. Finally, to analyze the captured images, Image J software was employed. The fibrosis percentage was determined by a blinded expert as a ratio of fibrotic area to the total pancreatic surface. In this way, the mice were classified as having under or over 50 % pancreatic fibrosis. In addition to the KC mice, five additional KPC (K-ras^LSL.G12D/+^; p53^LSL.R172H/+^; PdxCre^tg/+^) mice were also assessed. This transgenic mouse model develops PDAC after 5 months 51). Except for one TOP case, the remaining ones were BOP. High-resolution ultrasound imaging accompanied by weekly palpation helped in selecting them for scanning. The full-fledged tumor usually scanned when its diameter is between 3-5 mm 42, 43.

It should be noted that Picrosirius red (PSR) staining 52 is an established method used for fibrosis assessment because this method is capable of visualizing and quantifying the amount of collagen in tissue, reported as PSR density 53-55. As opposed to more traditional stains such as trichrome, Picrosirius red has selectivity that enables this method for both staining and quantification of collagen 55. The same procedure was applied to human pancreatic specimens.

### Young's modulus assessment of surgical human pancreatic specimens

In this study, human pancreatic ductal adenocarcinoma surgical resection specimens were examined by HME to estimate their local stiffness by calculating the corresponding Young's modulus. This study was first approved by the institutional review board of Columbia University, and all human studies were consented prior to participation.

The 33 specimens with PDAC tumor were received fresh from the Department of Pathology at Columbia University Irving Medical Center; one case was removed from this specimen population due to technical issue during the scan. There were a total of 32 surgically resected specimens with PDAC. Among these, 17 cases were resected through distal or total pancreatectomy and 15 cases were obtained through Whipple's procedure. The patient's age range was between 44 to 95 years old (68.8 ± 9.3 years old). The specimens were immersed in a degassed, PBS (Phosphate-Buffered Saline)-filled tank. An absorber was placed underneath the specimen to reduce reflection echoes.

Unlike the previous experiments where a single HMI focus mode was used, the human PDAC resection specimens were scanned in raster mode to cover the whole specimen. In each step, the 3D positioner was moved 3mm in axial and lateral directions and the overall Young's modulus 2D map was reconstructed based on the resulted Young's modulus at each step. The data acquisition was limited to 120 minutes in order to avoid autolysis and preserve the tissue for pathological examination.

During imaging of these freshly excised specimens, the probe was perpendicular to the pancreatic duct to have a solid correlation with the specimen slicing in pathology. In order to better understand the correlation between the structural changes, in particular the degree of fibrosis assessed histologically and the local stiffness as reported by the 2D YM map, after scanning, full-face sections of the pancreas corresponding to the HME plane were submitted for histological examination. Histological assessment of fibrosis was performed on the surgical human specimens for the tumor, its perilesional region and the normal surrounding tissue.

It should be noted that the acquired specimens were categorized in two groups. The first group contained 18 specimens with no prior treatment. The second group consisted of 14 specimens exposed to chemotherapy such as gemcitabine / Abraxane (GA), gemcitabine / Taxotere / Xeloda (GTX), FOLFIRINOX (FOL: Leucovorin Calcium (Folinic Acid), F: Fluorouracil, IRIN: Irinotecan Hydrochloride, OX: Oxaliplatin), with or without radiotherapy.

### Statistical Analysis

**One**-**way** analysis of variance (**ANOVA**) 56 was used to determine whether there are any statistically significant differences between the measured Young's Modulus (YM) for different regions within each human pancreatic specimen. Based on the measurements, three tissue regions were recognized and their stiffness measurements are statistically significant (p value = 0.05).These three regions are Tumor (YM > 40 kPa), Perilesional (10 kPa < YM < 30 kPa) and Non-neoplastic regions (YM < 10 kPa).

## Results

### *In vivo* Young's modulus assessment of PDAC solid tumors in transgenic mice

In this study, we used the genetically engineered mouse model Kras^LSL.G12D/+^; p53^LSL.R172H/+^; PdxCre^tg/+^ (KPC). This type of transgenic mouse model is a well-established and clinically-predictive *in vivo* model of PDAC 8, 43. In this pancreas-specific mouse model, the endogenous expression of point-mutant K-ras and p53 are responsible for cognate mutations, and both of them play a key role in the pathogenesis of PDAC in patients 8, 43. Newborn KPC mice did not show PDAC and were born with normal pancreata. However, gradually, over the course of several months, they developed pathological changes in the pancreas. Initially, they developed acinar-to-ductal metaplasia (ADM), followed by pancreatic intraepithelial neoplasia (PanIN) lesions and finally they progressed to overt ductal adenocarcinoma 43. These changes are accompanied by a progressive increase in parenchymal fibrosis. The fibrosis progression manifested as fibrotic extracellular matrix composition leads to elevation in the solid stress and interstitial fluid pressure (IFP) which may cause the changes observed in this phenotype 43. Thus, the KPC genetically engineered mouse model seemed a solid fit for our study to evaluate the HME performance in detecting fibrosis at different stages of progression by measuring stiffness noninvasively.

**Figure 2:**
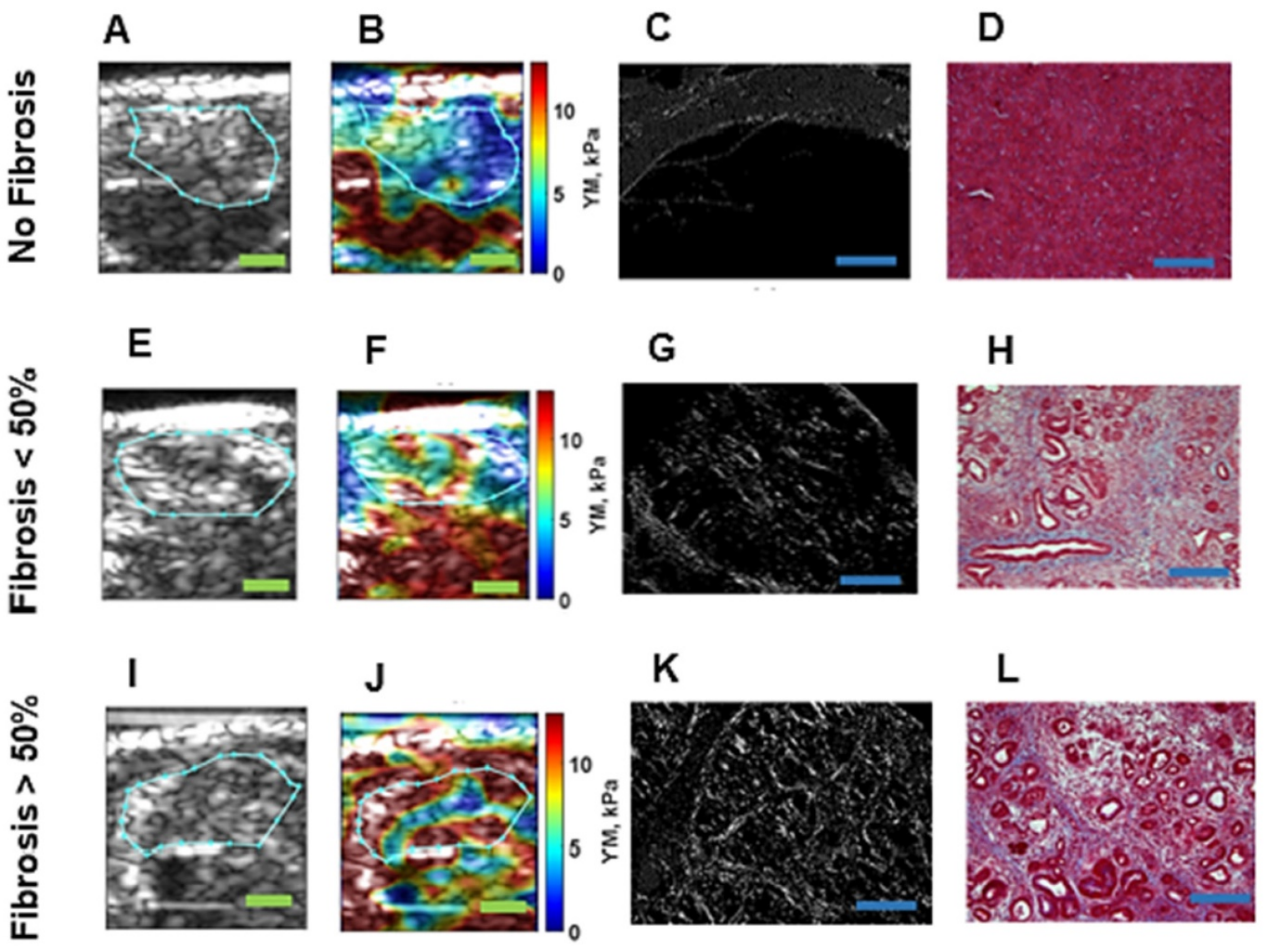
** Different fibrosis stages highlighted by Mason's trichrome with 20x magnification, Picrosirius red staining method, and the corresponding B-mode image and Young's modulus 2D maps overlaid on B-mode images. A:** B-mode image of pancreas, specified with cyan contour, with no fibrosis. **B:** 2D Young's modulus map of pancreas, specified with cyan contour, with no fibrosis overlaid on B-mode image the estimated median Young's modulus is **(YM = 3.6 kPa). C:** Picrosirius red slide of pancreas with no fibrosis **(PSR, density = 4.2%). D:** Mason's trichrome slide of pancreas with no fibrosis. **E:** B-mode image of pancreas, specified with cyan contour, with less than 50 % fibrosis. **F:** 2D Young's modulus map of pancreas, specified with cyan contour, with less than 50 % fibrosis overlaid on B-mode image. The estimated median Young's modulus is **(YM = 6.3 kPa). G:** Picrosirius red staining slide of pancreas with less than 50 % fibrosis **(PSR, density = 7.1%).** Mason's trichrome slide of pancreas with less than 50 % fibrosis. **H:** Mason's trichrome slide of pancreas with less than 50 % fibrosis. **I:** B-mode image of pancreas, specified with cyan contour, with more than 50 % fibrosis. **J:** 2D Young's modulus map of pancreas, specified with cyan contour, with more than 50 % fibrosis overlaid on B-mode image. The estimated median Young's modulus is **(YM = 11 kPa)** specified with cyan contour. **K:** Picrosirius red slide of pancreas with more than 50 % fibrosis. The Picrosirius density is **(PSR, density = 12.1 %). L:** Mason's trichrome slide of pancreas with more than 50 % fibrosis. Scale bar 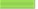 for B-mode and Young's modulus 2D maps are 2 mm and for Masson's trichrome and Picrosirius images, 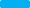 are 200µm.

We applied HME *in vivo* to these mice with different levels of pancreatic fibrosis, including mice with normal pancreas, mice with < 50 % fibrosis, mice with > 50 % fibrosis and finally mice with pancreatic tumors. Figure 2 demonstrates the result of this study in mice representative for each group. The corresponding histological images accompanied by 2D Young's modulus map for each mouse is depicted. We calculated the median value of the YM in each murine pancreas based on the selected ROI in each 2D Young's modulus map, as mentioned in the caption of Figure 2. In the caption of this figure, the PSR density (collagen density) and the Young's modulus corresponding to each case are documented.

In cases with no fibrosis, the acinar cells are the dominant part of the pancreatic tissue, as depicted in Figure 2D 57, 58. (YM = 3.6 kPa, PSR = 4.2 %). For samples with fibrosis under 50 %, the proportion of acinar cells appears decreased, and the amount of fibrosis is increased. The loss of pancreatic acinar cells and epithelial atrophy are seen in Figure 2H, 57, 58. (YM = 6.3 kPa, PSR = 7.1 %). When fibrosis is > 50 %, the fibrosis is predominant, fewer acinar cells are visible and the residual epithelium appears atrophic, as seen in the corresponding Masson's trichrome in Figure 2L 57, 58. (YM = 11 kPa, PSR = 12.1 %). YM is much higher than in the previously displayed cases with less than 50% fibrosis. It is also worth mentioning that in Figure 2E and F the organ under the pancreas is the kidney, which is known to be stiffer 42.

**Figure 3:**
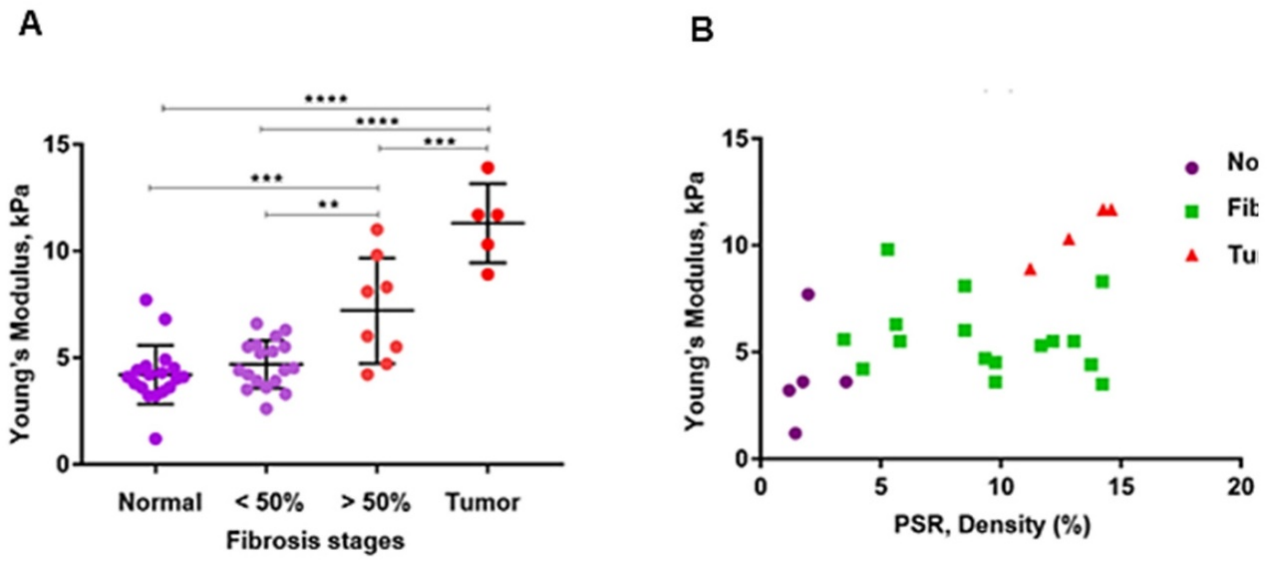
** A:** The estimated median Young's modulus measured *in vivo* in normal murine pancreas (n = 18). **YM = (4.2 ± 1.3) kPa**, in non-neoplastic pancreas with less than 50 % fibrosis (n = 18). **YM = (4.7 ± 1.1) kPa,** in non-neoplastic pancreas with more than 50% fibrosis (n = 8). **YM = (7.2 ± 2.3) kPa,** and in pancreatic tumors (n = 5). **YM = (11.3 ± 1.7) kPa.** (One-Way ANOVA: **P < 0.0025, ***P < 0.0003, ****P < 0.0001). **B:** Estimated median Young's modulus values vs. PSR density percentage using Picrosirius red staining. This method was applied on 5 of the total normal pancreata, **PSR= (2 ± 0.8) %**, on 17 of the non-neoplastic fibrotic pancreatitis cases, **PSR = (9.8 ± 3.4) %,** and on 4 with PDAC, **PSR = (13.2 ± 1.2) %.**

Figure 3A, B and Table 1 summarize the HME measurements, i.e., the Young's moduli for different levels of pancreatic fibrosis, as previously mentioned. Figure 3A shows the HME measurements in mice including normal pancreata, (n = 18), fibrotic pancreata consisting of chronic pancreatitis with less than 50 % fibrosis, (n=18), and more than 50% fibrosis, (n=8). Thus, the overall number of fibrotic pancreata is 26, (n = 26). Finally the number of mice with PDAC is 5, (n=5). Figure 3B shows the HME and PSR (%) mouse results. For normal pancreata 5 of 18 cases, for fibrotic pancreata 17 of 26 and for PDAC 4 of 5 mice were studied by HME and PSR.

Table 1 lists the average and standard deviation of the median Young's modulus and PSR density (%) for the mice undergoing both HME and PSR was shown in Figure 3B.

### Young's modulus assessment of PDAC solid tumors in surgical human specimen

**Figure 4:**
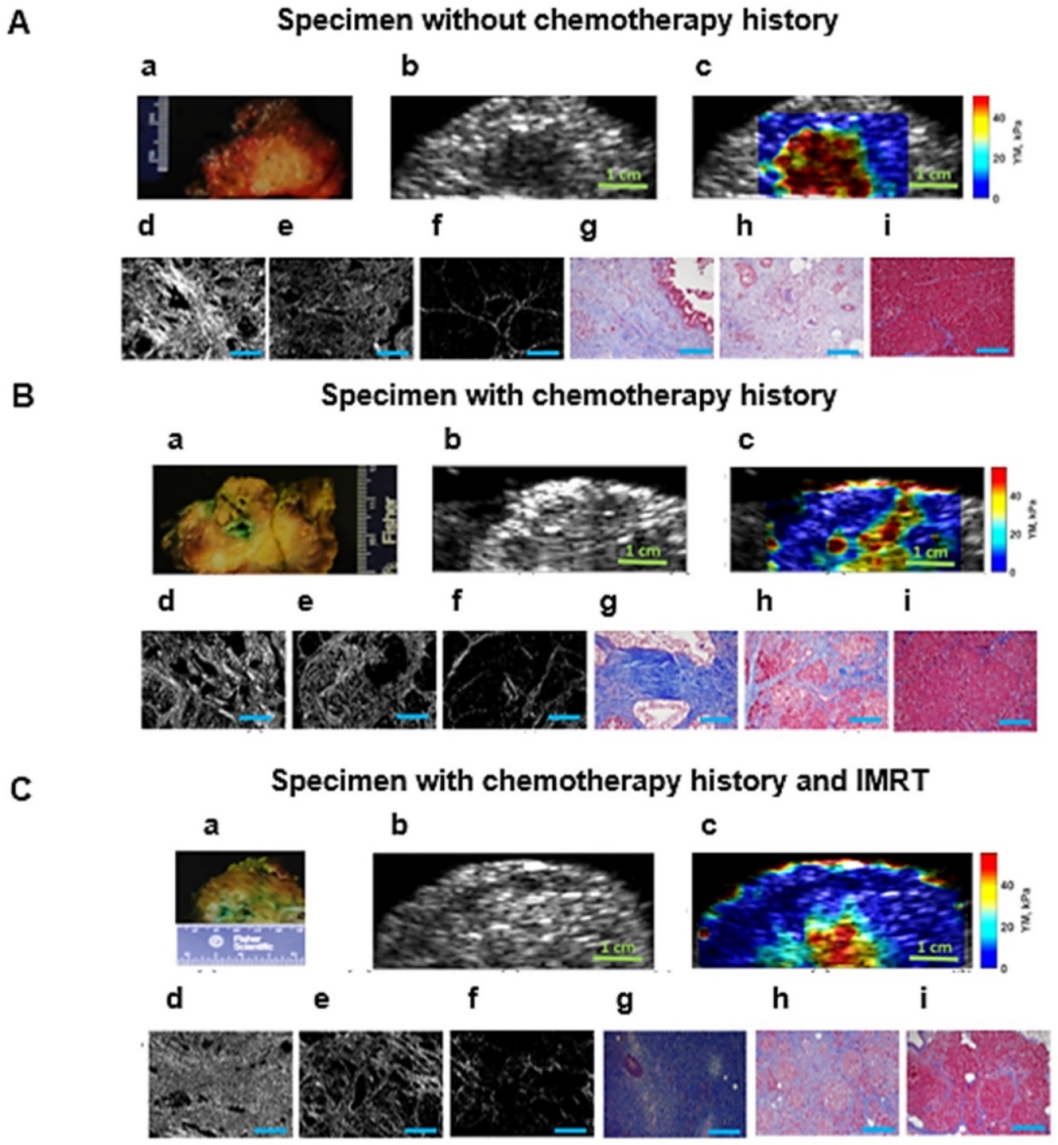
** A: Surgical pancreatic human specimens. a:** Gross photograph of PDAC tumor and its surrounding tissue in cross section. **b:** B-mode image of PDAC tumor and its surrounding tissue. **c:** 2D Young's modulus map overlaid on B-mode image. The estimated median Young's modulus for tumor part, red/orange/yellow part is **(YM = 44.9 kPa).** The estimated median Young's modulus for perilesional part, light blue part, is **(YM = 19.2 kPa).** The estimated median Young's modulus for non-neoplastic part, dark blue part is **(YM = 3.8 kPa). d:** Picrosirius red stain of a PDAC tumor, 20x magnification **(PSR, density = 53.2 %). e:** Picrosirius red stain of the perilesional region surrounding the PDAC tumor, 20x magnification **(PSR, density = 35 %). f:** Picrosirius red stain of the non-neoplastic pancreas adjacent to PDAC tumor, 20x magnification **(PSR, density = 4.6 %). g:** Mason's trichrome stain of PDAC tumor, 20x magnification. **h:** Mason's trichrome stain of the perilesional region of PDAC tumor, 20x magnification. **i:** Mason's trichrome stain of non-neoplastic pancreas adjacent to PDAC tumor, 20x magnification. **B: Human surgical pancreatic specimens with history of neoadjuvant treatment. (Gemcitabin / Abraxane, 6 months). a:** Gross photograph of PDAC tumor, status post neoadjuvant treatment, and its surrounding tissue in cross section. **b:** B-mode image of PDAC status post neoadjuvant treatment and its surrounding tissue. **c:** 2D Young's modulus map overlaid on B-mode image. The estimated median Young's modulus for tumor part, red/orange/yellow area, is **(YM = 35.3 kPa).** The estimated median Young's modulus for the perilesional region, light blue part, is **(YM = 18 kPa).** The estimated median Young's modulus for non-neoplastic part, dark blue part is **(YM = 3.1 kPa). d:** Picrosirius red stain of PDAC tumor status post neoadjuvant treatment, 20x magnification **(PSR, density = 47.1 %). e:** Picrosirius red stain of the perilesional region of PDAC tumor, 20x magnification **(PSR, density = 35.4 %). f:** Picrosirius red stain of non-neoplastic pancreas adjacent to PDAC tumor, 20x magnification **(PSR, density = 6.6 %). g:** Mason's trichrome stain of PDAC tumor status post neoadjuvant treatment, 20x magnification. **h:** Mason's trichrome stain of the perilesional region of PDAC tumor status post neoadjuvant treatment, 20x magnification. **i:** Mason's trichrome stains of the non-neoplastic pancreas adjacent to PDAC tumor, 20x magnification. **C: Human surgical pancreatic specimens with history of neoadjuvant treatment**. Chemo/radiation (Gemcitabin /Abraxane, 3 months, accompanied by IMRT intensity-modulated radiation therapy of 50.4 Gy)) **a:** Gross photograph of PDAC tumor and its surrounding tissue in cross section. **b:** B-mode image of PDAC and its surrounding tissue. **c:** 2D Young's modulus map overlaid on B-mode image. The estimated median Young's modulus for tumor part, red/orange/yellow part, is **(YM = 40 kPa).** The estimated median Young's modulus for perilesional region of PDAC tumor, light blue area, is **(YM =17.2 kPa).** The estimated median Young's modulus for the non-neoplastic pancreas adjacent to PDAC tumor, dark blue, is **(YM =2.9 kPa). d:** Picrosirius red stain of PDAC tumor status post neoadjuvant treatment, 20x magnification **(PSR, density = 57 %). e:** Picrosirius red stain of the perilesional region of a PDAC tumor status post neoadjuvant treatment, 20x magnification **(PSR, density = 23.6 %). f:** Picrosirius red stain of non-neoplastic pancreas adjacent to a PDAC tumor status post neoadjuvant treatment, 20x magnification **(PSR, density = 5 %). g:** Mason's trichrome stain of PDAC tumor status post neoadjuvant treatment, 10x magnification. **h:** Mason's trichrome stain of the perilesional region of a PDAC tumor status post neoadjuvant treatment, 10x magnification. **i:** Mason's trichrome stain of non-neoplastic pancreas adjacent to a PDAC tumor status post neoadjuvant treatment, 10x magnification. Scale bars for Masson's trichrome and Picrosirius stain images displayed as 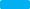 are equal to 200µm.

In this study we show that for the specimens in all categories we can differentiate between tumors, perilesional and non-neoplastic (normal) areas surrounding the pancreatic tumor based on the significant differences in their estimated Young's modulus. Figure 4 illustrates the HME method application on surgical specimens from patients with no chemotherapy treatment, and with neoadjuvant chemotherapy and radiotherapy. In addition, the Mason's trichrome stain and Picrosirius red stain of various parts of the specimen including tumor, perilesional and non-neoplastic regions are shown. As we have explained before, the Picrosirius red method can quantify the fibrosis amount. In the caption of each figure the estimated corresponding Young's modulus of the specified part, and the corresponding PSR density percentage are indicated. As these aforementioned results showed, the reported Young's modulus values for all cases were concordant with the Picrosirius red methodology for fibrosis assessment as an independent microscopic method. In other words, the estimated Young's modulus increased with collagen density PSR (%). However, the size of the perilesional region is variable as you can see in this figure (Fig. 4). It depends on the size of the tumor, treatment history and the tumor stage.

**Figure 5:**
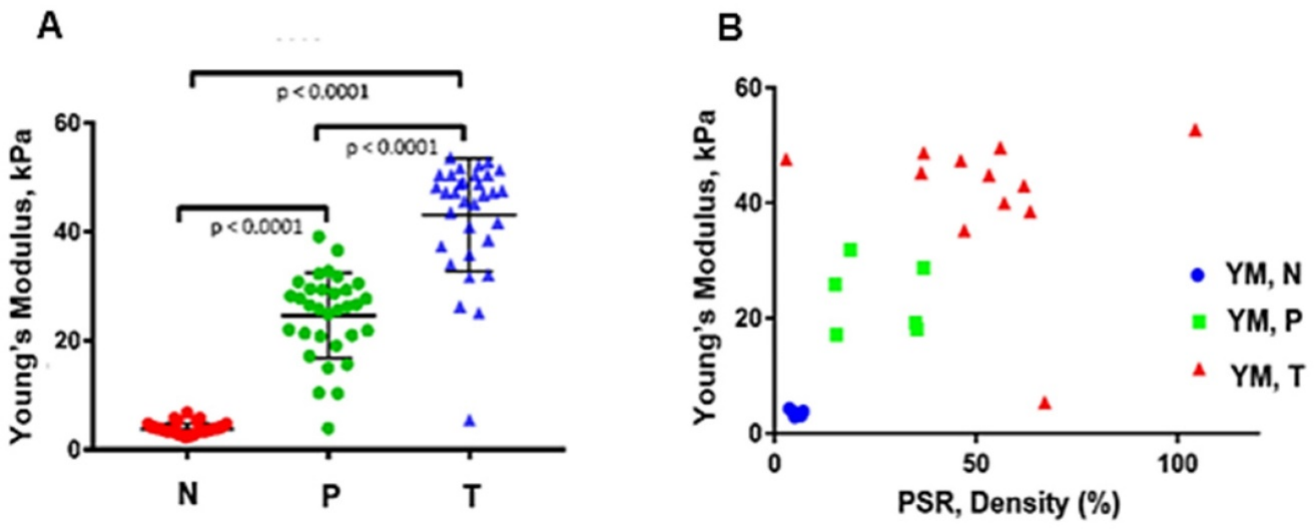
** A:** The median Young's modulus estimation of all PDAC surgical specimens (n=32) regardless of their history of therapy (One-Way ANOVA). **N:** Non-neoplastic regions, with mean and standard deviation of: **YM = (4 ± 1.6) kPa. P:** Perilesional region, **YM = (23.9 ± 8) kPa**. **T:** Tumor, **YM = (42.9 ± 10.2) kPa.** See text for details.** B:** Estimated median Young's modulus values vs. PSR density (%) using Picrosirius red staining of post-surgical human specimens. The number of **T**: tumor samples using PSR analysis are n = 11 with mean and standard deviation of **(51.4 ± 23.3) %,** for perilesional samples,** P**, using PSR analysis, n = 6 with mean and standard deviation of **(26.1 ± 9.8) %,** and the number of none-neo plastic or normal samples,** N**, using PSR on them, n = 7 with mean and standard deviation **(5.3 ± 1.1)%.**

In order to observe the relationship between measured stiffness, YM, using the HME method, and fibrosis progression, we applied the Picrosirius red staining method on tumor, perilesional, and non-neoplastic regions of the surgical human specimens. The results are illustrated in Figure 5A and B. Moreover, Table 3 further details these results.

Table 2 shows the characteristics of the patients in our study, including gender, age, tumor site, type of surgery and neoadjuvant treatment history. Chemotherapy included gemcitabine / Abraxane (GA), gemcitabine / Taxotere / Xeloda (GTX), FOLFIRINOX, FFX (FOL: Leucovorin Calcium (Folinic Acid), F: Fluorouracil, IRIN: Irinotecan Hydrochloride, OX: Oxaliplatin). Radiotherapy consisted of SBRT (Stereotactic Body Radiation Therapy) and IMRT (Intensive Modulated Radiation Therapy).

## Discussion

Development of extremely fibrotic stroma in PDAC tumors is due to the dense and cross-linked extracellular matrix (ECM) formation that occurs as the disease progresses 6. Fibrosis promotes stiffness elevation in stroma and the newly developed rigid stroma promotes a tensional homeostasis that culminates in a high level of cell contractility 6, 21, 59, 60.

In this study, we first showed that the stiffness values that we measured by the HME method were in the range of the values that have been reported by using the previously reported shear wave methods 37-41. Although these current elastography methods have been used for stiffness measurements of pancreas, none of them reported the Young's modulus value of perilesional region within the specimen.

In our animal study, we evaluated the performance of the HME method 36 in assessing pancreatic tumors in transgenic mice with various levels of fibrosis. The results showed strong correlation between the reported Young's modulus value using HME and the fibrosis level seen on histologic examination.

Figures 3 and 5 summarize the mouse and surgical human specimen's findings, showing a strong correlation between the measured Young's modulus using HME and collagen density using microscopic PSR method. More importantly, these figures and Table 3 demonstrate that HME and Young's modulus assessment facilitated recognition of three different regions in human specimens: non-neoplastic region, N, perilesional region, P, and tumor region, T. This capability of HME could assist surgical planning in delineating tumor boundaries intraoperatively.

This could help reduce positive surgical margins, increase complete tumor resection rates, and therefore, potentially reduce recurrence rates for this aggressive neoplasm 12, 60. It should be noted that, to the best of our knowledge, there are no criteria described so far to estimate the perilesional area's stiffness (Young's modulus) separately prior to this work. This method, HME, could help the surgeons to delineate the tumors more accurately prior and during the surgery.

Application of two independent methods, PSR and HME on both transgenic mice with different fibrosis levels and in human PDAC specimens had great advantages. For instance, the range of stiffness change in the former group was much lower than in the latter one. This meant that although in transgenic mice the range of stiffness alteration is much lower comparing to human specimens, both collagen density, i.e., PSR density (%), and HME were sensitive enough to detect this lower stiffness change in the range of 4 kPa to 12 kPa and fibrosis rate, PSR %, in the range of 2 % to 14 % respectively, Table 1. In the human specimens, the contrasts were much greater for these measured values, Table 3. However, even smaller stiffness changes at the border of the tumors can be detected by HME, in the range of 4 to 12 kPa as we show in this animal study.

Another advantage of this study was that HME was applied on both *ex vivo* and *in vivo* cases. Thus, the complexity of the *in vivo* study had no significant effects on HME Young's modulus measurement. This was due to HME characteristics such as its generated AM frequency deformation in tissue and its constant harmonic nature 36.

In this study we also showed that there was a significant difference between the measured Young's modulus values of PDAC tumors in transgenic mice (YM < 14 kPa) and in human specimens (YM < 60 kPa). As we demonstrated in this study, the HME Young's modulus estimation of PDAC tumors corroborated with previous studies using different mechanical testing for Young's modulus estimation in transgenic mice 6. Similarly, the same trend was obtained in human cases 38-41. In mice studies, the PSR density percentage for normal pancreas was (2 ± 0.8) %, for fibrotic cases (KC) was (9.8 ± 3.4) %, and for cases with full-fledged tumor (KPC), PSR density was (13.2 ± 1.2) %, (Figure 3 A, B and Table 1). In human specimens, PSR density was (5.3 ± 1.1) % for non-neoplastic, (26.1 ± 9.8) % for perilesional, and (51.4 ± 23.3) % for tumor (Figure 5 A, B, and Table 3).

**Figure 6:**
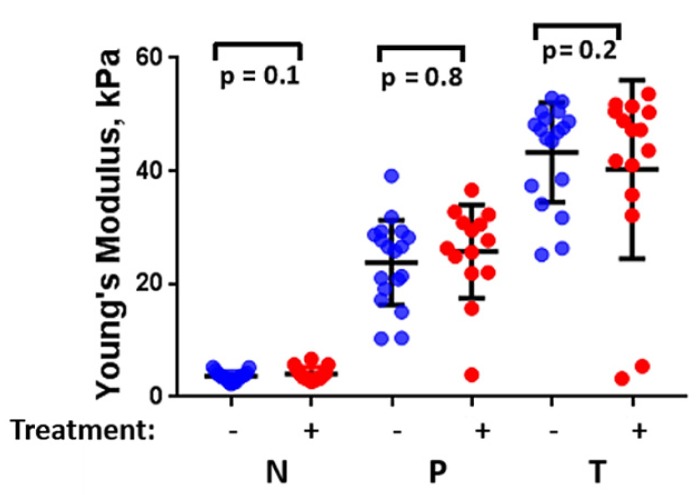
The estimated median Young's modulus value of all PDAC surgical specimens (n=32) considering their chemotherapy history (with chemotherapy history (+), without chemotherapy history (-). (One-Way ANOVA). **N:** Non-neoplastic, **P:** Perilesional region, **T:** Tumor region.

Statistical analysis of these pancreatic specimens including both groups, with and without chemotherapy history, as shown in Figure 6, indicates that there is no significant difference in the measured stiffness between treated and untreated samples.

As part of our future work, we will focus on intraoperative application of HME in order to assist surgeons to perform tumor resection more accurately with the ultimate goal of reduction in recurrence rate 12, 60. Moreover, monitoring tumor stiffness may better inform the decision of a timely surgical intervention 9-12, 60. In addition, other factors such as tumor cellularity and the ratio of epithelial to stroma components may play a role. With further understanding of the correlation between non-neoplastic tissue structure and stiffness, the HME method could be used as a prognostic imaging method in patients with PDAC to evaluate the efficiency of their treatment.

As we emphasized previously, the main advantage of using the HME method is represented by its capability to differentiate between the tumor, perilesional, and adjacent non-neoplastic region within each specimen by measuring the Young's modulus and generating the stiffness 2D map in which these three region are clearly specified. This is a unique advantage of the HME method. The current technologies use just one imaging transducer to apply the radiation force and record the data 30-34, 37-41. Thus, adjusting the duration and amplitude of the radiation force is much more limited comparing to the HME method in which one transducer is for the radiation force and the other one is used to collect the data. In addition, the harmonic nature of the radiation force generated in HME compared to the multiple pulses used in previous shear wave methods makes HME more resistant to shear wave attenuation that is a challenge for the majority of the previously described shear wave methods. In addition, the raster scanning used in this technique helps in generating the high resolution 2D Young's modulus maps.

In spite of the all aforementioned advantages of HME, in some of the generated 2D Young's modulus maps like Figures 4B, c and 4C, c there is an overestimation of the Young's modulus estimation at the boundary of the specimen 36. The Picrosirius red stained sections were generated from the same region that was scanned for the 2D Young's modulus map. Due to difference in scale, matching region where the microscopic images were generated was challenging and is of the limitations of the study. In addition, with this method data acquisition may take longer in raster scanning. Moreover, the HME setting is larger and needs more electronics compared to current shear wave methods.

## Conclusion

This study showed that increasing fibrosis in murine PDAC tumors was associated with elevation in both collagen density measured by Picrosirius red staining and Young's modulus quantification of tumor stiffness. We observed a similar trend in each individual pancreatic human specimen. Thus, three different regions were identified. The tumor region showed the highest collagen density, % PSR, while the lowest measured PSR value corresponded to the non-neoplastic region. In addition, the measured PSR values related to perilesional regions fell in intermediate range. 2D Young's modulus maps generated by HME of each human specimen showed a similar trend. In summary, HME has the capability of generating 2D Young's modulus maps non-invasively. This method may provide new avenues for detecting and staging of PDAC tumors based on the collagen content, assessing tumor response to chemotherapy and resectability.

## Figures and Tables

**Table 1:** Summary of applying HME and Picrosirius red method on transgenic mice

Pancreta type	Number	Young's modulus (HME), kPa	PSR density (%)
Normal	5	4.2 ± 1.3	2.0 ± 0.8
Fibrotic	17	5.5 ± 2.0	9.8 ± 3.4
PDAC	4	11.3 ± 1.7	13.2 ± 1.2

**Table 2:** Patient population and characteristics

Case #	Gender	Age	Tumor Site	Type of Surgery	Neoajuvant Therapy
1	F	68	Body/Tail	Distal pancreatectomy	No prior therapy
2	F	67	Body	Distal pancreatectomy	No prior therapy
3	M	67	Body	Distal pancreatectomy	4 months GTX + SBRT
4	M	67	Body	Distal pancreatectomy	No prior therapy
5	F	62	Tail	Distal pancreatectomy	No prior therapy
6	F	95	Head	Total pancreatectomy	No prior therapy
7	M	70	Head	Whipple procedure	4 months GTX + SBRT
8	F	73	Body	Distal pancreatectomy	2 months of gemcitabine-based chemo
9	M	73	Head	Whipple procedure	3 months of gemcitabine/Abraxane + IMRT
10	F	82	Head	Whipple procedure	6 months gemcitabine/Abraxane
11	F	62	Head	Whipple procedure	No prior therapy
12	M	82	Head	Whipple procedure	No prior therapy
13	M	58	Head	Whipple procedure	4 months FOLFIRINOX + SBRT
14	M	71	Head	Whipple procedure	No prior therapy
15	M	72	Head	Whipple procedure	No prior therapy
17	M	65	n/a	Pancreatectomy	No prior therapy
18	M	63	n/a	Distal pancreatectomy	Gemcitabine for short time
19	F	71	n/a	Distal pancreatectomy	Chemo(GTX)
20	F	44	n/a	Pancreatectomy	Chemo(Gem)
21	F	80	n/a	Distal pancreatectomy	No prior therapy
22	F	65	n/a	Distal pancreatectomy	
23	M	70	n/a	Distal pancreatectomy	No prior therapy
24	F	54	Head	Whipple procedure	No prior therapy
25	F	68	Head	Whipple procedure	FFX
26	F	64	Head	Whipple procedure	No prior therapy
27	F	77	Head	Whipple procedure	
28	F	76	n/a	Distal pancreatectomy	No prior therapy
29	F	53	n/a	Distal pancreatectomy	Chemo (FFX), SBRT
30	F	65	Head	Whipple procedure	FFX, SBRT
31	M	73	Head	Whipple procedure	
32	M	72		Distal pancreatectomy	Chemo ( FFX ) + SBRT
33	F	74	Head	Whipple procedure	FFX

**Table 3:** Summary of applying both HME and Picrosirius red method on pancreatic human specimens

Region	Number of specimens	Young's modulus (HME), kPa	PSR density (%)
None-neoplastic, N	7	3.5 ± 0.5	5.3 ± 1.1
Perilesional, P	6	23.5 ± 5.6	26.1 ± 9.8
Tumor, T	11	44.8 ± 5	51.4 ± 23.3

## References

[1] Vincent A, Herman J, Schulick R, Hruban RH, Goggins M (2011). Pancreatic cancer. The lancet.

[2] Hidalgo M, Cascinu S, Kleeff J, Labianca R, Löhr J-M, Neoptolemos J (2015). Addressing the challenges of pancreatic cancer: future directions for improving outcomes. Pancreatology.

[3] Siegel R, Naishadham D, Jemal A (2012). Cancer statistics for hispanics/latinos, 2012. CA: a cancer journal for clinicians.

[4] Rahib L, Smith BD, Aizenberg R, Rosenzweig AB, Fleshman JM, Matrisian LM (2014). Projecting cancer incidence and deaths to 2030: the unexpected burden of thyroid, liver, and pancreas cancers in the United States. Cancer research.

[5] Ferlay J, Partensky C, Bray F (2016). More deaths from pancreatic cancer than breast cancer in the EU by 2017. Acta Oncologica.

[6] Rice A, Cortes E, Lachowski D, Cheung B, Karim S, Morton J (2017). Matrix stiffness induces epithelial-mesenchymal transition and promotes chemoresistance in pancreatic cancer cells. Oncogenesis.

[7] Ardito CM, Grüner BM, Takeuchi KK, Lubeseder-Martellato C, Teichmann N, Mazur PK (2012). EGF receptor is required for KRAS-induced pancreatic tumorigenesis. Cancer cell.

[8] Olive KP, Jacobetz MA, Davidson CJ, Gopinathan A, McIntyre D, Honess D (2009). Inhibition of Hedgehog signaling enhances delivery of chemotherapy in a mouse model of pancreatic cancer. Science.

[9] Bockhorn M, Uzunoglu FG, Adham M, Imrie C, Milicevic M, Sandberg AA (2014). Borderline resectable pancreatic cancer: a consensus statement by the International Study Group of Pancreatic Surgery (ISGPS). Surgery.

[10] Al-Hawary MM, Francis IR, Chari ST, Fishman EK, Hough DM, Lu DS (2014). Pancreatic ductal adenocarcinoma radiology reporting template: consensus statement of the Society of Abdominal Radiology and the American Pancreatic Association. Radiology.

[11] Morgan DE, Waggoner CN, Canon CL, Lockhart ME, Fineberg NS, Posey III JA (2010). Resectability of pancreatic adenocarcinoma in patients with locally advanced disease downstaged by preoperative therapy: a challenge for MDCT. American Journal of Roentgenology.

[12] Kluger MD, Rashid MF, Rosario VL, Schrope BA, Steinman JA, Hecht EM (2018). Resection of locally advanced pancreatic cancer without regression of arterial encasement after modern-era neoadjuvant therapy. Journal of Gastrointestinal Surgery.

[13] Jacobetz MA, Chan DS, Neesse A, Bapiro TE, Cook N, Frese KK (2013). Hyaluronan impairs vascular function and drug delivery in a mouse model of pancreatic cancer. Gut.

[14] Nia H, Liu H, Seano G, Datta M, Jones D, Rahbari N (2017). Solid stress and elastic energy as measures of tumor mechanopathology. AACR.

[15] Erkan M, Reiser-Erkan C, Michalski CW, Deucker S, Sauliunaite D, Streit S (2009). Cancer-stellate cell interactions perpetuate the hypoxia-fibrosis cycle in pancreatic ductal adenocarcinoma. Neoplasia.

[16] Conroy T, Desseigne F, Ychou M, Bouché O, Guimbaud R, Bécouarn Y (2011). FOLFIRINOX versus gemcitabine for metastatic pancreatic cancer. New England Journal of Medicine.

[17] Yip D, Karapetis C, Strickland A, Steer CB, Goldstein D (2006). Chemotherapy and radiotherapy for inoperable advanced pancreatic cancer. Cochrane Database Syst Rev.

[18] Conroy T, Hammel P, Hebbar M, Ben Abdelghani M, Wei AC-c, Raoul J-L (2018). Unicancer GI PRODIGE 24/CCTG PA. 6 trial: A multicenter international randomized phase III trial of adjuvant mFOLFIRINOX versus gemcitabine (gem) in patients with resected pancreatic ductal adenocarcinomas. American Society of Clinical Oncology.

[19] Von Hoff DD, Ervin T, Arena FP, Chiorean EG, Infante J, Moore M (2013). Increased survival in pancreatic cancer with nab-paclitaxel plus gemcitabine. New England Journal of Medicine.

[20] Wynn TA (2008). Cellular and molecular mechanisms of fibrosis. J Pathol.

[21] Imamura T, Iguchi H, Manabe T, Ohshio G, Yoshimura T, Wang Z (1995). Quantitative analysis of collagen and collagen subtypes I, III, and V in human pancreatic cancer, tumor-associated chronic pancreatitis, and alcoholic chronic pancreatitis. Pancreas.

[22] Mollenhauer J, Roether I, Kern H (1987). Distribution of extracellular matrix proteins in pancreatic ductal adenocarcinoma and its influence on tumor cell proliferation *in vitro*. Pancreas.

[23] Linder S, Castanos-Velez E, Biberfeld P (2001). Immunohistochemical expression of extracellular matrix proteins and adhesion molecules in pancreatic carcinoma. Hepato-gastroenterology.

[24] Olivares O, Mayers JR, Gouirand V, Torrence ME, Gicquel T, Borge L (2017). Collagen-derived proline promotes pancreatic ductal adenocarcinoma cell survival under nutrient limited conditions. Nature communications.

[25] Whatcott CJ, Diep CH, Jiang P, Watanabe A, LoBello J, Sima C (2015). Desmoplasia in primary tumors and metastatic lesions of pancreatic cancer. Clinical Cancer Research.

[26] Baker A, Bird D, Lang G, Cox TR, Erler J (2013). Lysyl oxidase enzymatic function increases stiffness to drive colorectal cancer progression through FAK. Oncogene.

[27] Elyas E, Papaevangelou E, Alles EJ, Erler JT, Cox TR, Robinson SP (2017). Correlation of ultrasound shear wave elastography with pathological analysis in a xenografic tumour model. Scientific reports.

[28] Li G-Y, Cao Y (2017). Mechanics of ultrasound elastography. Proceedings of the Royal Society A: Mathematical, Physical and Engineering Sciences.

[29] Sarvazyan A, J Hall T, W Urban M, Fatemi M, R Aglyamov S, S Garra B (2011). An overview of elastography-an emerging branch of medical imaging. Current medical imaging reviews.

[30] Sarvazyan AP, Rudenko OV, Swanson SD, Fowlkes JB, Emelianov SY (1998). Shear wave elasticity imaging: a new ultrasonic technology of medical diagnostics. Ultrasound in medicine & biology.

[31] Bercoff J, Tanter M, Fink M (2004). Supersonic shear imaging: a new technique for soft tissue elasticity mapping. IEEE transactions on ultrasonics, ferroelectrics, and frequency control.

[32] Nightingale K, McAleavey S, Trahey G (2003). Shear-wave generation using acoustic radiation force: *in vivo* and *ex vivo* results. Ultrasound in medicine & biology.

[33] Chen S, Urban MW, Pislaru C, Kinnick R, Zheng Y, Yao A (2009). Shearwave dispersion ultrasound vibrometry (SDUV) for measuring tissue elasticity and viscosity. IEEE transactions on ultrasonics, ferroelectrics, and frequency control.

[34] Song P, Zhao H, Manduca A, Urban MW, Greenleaf JF, Chen S (2012). Comb-push ultrasound shear elastography (CUSE): a novel method for two-dimensional shear elasticity imaging of soft tissues. IEEE transactions on medical imaging.

[35] Maleke C, Konofagou EE (2008). Harmonic motion imaging for focused ultrasound (HMIFU): a fully integrated technique for sonication and monitoring of thermal ablation in tissues. Physics in Medicine & Biology.

[36] Nabavizadeh A, Payen T, Saharkhiz N, McGarry M, Olive KP, Konofagou EE (2018). *In vivo* Young's modulus mapping of pancreatic ductal adenocarcinoma during HIFU ablation using harmonic motion elastography (HME). Medical physics.

[37] D'Onofrio M, De Robertis R, Crosara S, Poli C, Canestrini S, Demozzi E (2016). Acoustic radiation force impulse with shear wave speed quantification of pancreatic masses: A prospective study. Pancreatology.

[38] Kawada N, Tanaka S, Uehara H, Ohkawa K, Yamai T, Takada R (2014). Potential use of point shear wave elastography for the pancreas: a single center prospective study. European journal of radiology.

[39] Yashima Y, Sasahira N, Isayama H, Kogure H, Ikeda H, Hirano K (2012). Acoustic radiation force impulse elastography for noninvasive assessment of chronic pancreatitis. Journal of gastroenterology.

[40] Anvari A, Barr RG, Dhyani M, Samir AE (2015). Clinical application of sonoelastography in thyroid, prostate, kidney, pancreas, and deep venous thrombosis. Abdominal imaging.

[41] Whatcott CJ, Diep CH, Jiang P, Watanabe A, LoBello J, Sima C (2015). Desmoplasia in primary tumors and metastatic lesions of pancreatic cancer. Clinical Cancer Research.

[42] Payen T, Palermo CF, Sastra SA, Chen H, Han Y, Olive KP (2016). Elasticity mapping of murine abdominal organs *in vivo* using harmonic motion imaging (HMI). Physics in Medicine & Biology.

[43] Payen T, Oberstein P, Saharkhiz N, Palermo CF, Sastra SA, Han Y (2018). Harmonic Motion Imaging of pancreatic tumor stiffness indicates disease state and treatment. Clin Cancer Res.

[44] Hou GY, Provost J, Grondin J, Wang S, Marquet F, Bunting E (2014). Sparse matrix beamforming and image reconstruction for 2-D HIFU monitoring using harmonic motion imaging for focused ultrasound (HMIFU) with *in vitro* validation. IEEE transactions on medical imaging.

[45] Luo J, Konofagou EE (2010). A fast normalized cross-correlation calculation method for motion estimation. IEEE transactions on ultrasonics, ferroelectrics, and frequency control.

[46] Manduca A, Lake DS, Kruse SA, Ehman RL (2003). Spatio-temporal directional filtering for improved inversion of MR elastography images. Medical image analysis.

[47] Deffieux T, Gennisson J-L, Bercoff J, Tanter M (2011). On the effects of reflected waves in transient shear wave elastography. IEEE transactions on ultrasonics, ferroelectrics, and frequency control.

[48] Song P, Manduca A, Zhao H, Urban MW, Greenleaf JF, Chen S (2014). Fast shear compounding using robust 2-D shear wave speed calculation and multi-directional filtering. Ultrasound in medicine & biology.

[49] Brembeck FH, Schreiber FS, Deramaudt TB, Craig L, Rhoades B, Swain G (2003). The mutant K-ras oncogene causes pancreatic periductal lymphocytic infiltration and gastric mucous neck cell hyperplasia in transgenic mice. Cancer research.

[50] Riegler J, Labyed Y, Rosenzweig S, Javinal V, Castiglioni A, Dominguez CX (2018). Tumor elastography and its association with collagen and the tumor microenvironment. Clinical Cancer Research.

[51] Hingorani SR, Wang L, Multani AS, Combs C, Deramaudt TB, Hruban RH (2005). Trp53R172H and KrasG12D cooperate to promote chromosomal instability and widely metastatic pancreatic ductal adenocarcinoma in mice. Cancer cell.

[52] Hadi AM, Mouchaers KT, Schalij I, Grunberg K, Meijer GA, Vonk-Noordegraaf A (2011). Rapid quantification of myocardial fibrosis: a new macro-based automated analysis. Cellular oncology.

[53] Junqueira LCU, Bignolas G, Brentani RR (1979). Picrosirius staining plus polarization microscopy, a specific method for collagen detection in tissue sections. The Histochemical journal.

[54] Puchtler H, Waldrop FS, Valentine LS (1973). Polarization microscopic studies of connective tissue stained with picro-sirius red FBA. Beiträge zur Pathologie.

[55] Whittaker P, Kloner R, Boughner D, Pickering J (1994). Quantitative assessment of myocardial collagen with picrosirius red staining and circularly polarized light. Basic research in cardiology.

[56] Howell DC (2009). Statistical methods for psychology. Cengage Learning.

[57] Chen K, Qian W, Jiang Z, Cheng L, Li J, Sun L (2017). Metformin suppresses cancer initiation and progression in genetic mouse models of pancreatic cancer. Molecular cancer.

[58] Wang LM, Silva MA, D'Costa Z, Bockelmann R, Soonawalla Z, Liu S (2016). The prognostic role of desmoplastic stroma in pancreatic ductal adenocarcinoma. Oncotarget.

[59] Nabavizadeh A, Bayat M, Kumar V, Gregory A, Webb J, Alizad A (2019). Viscoelastic biomarker for differentiation of benign and malignant breast lesion in ultra-low frequency range. Scientific reports.

[60] Ethun CG, Kooby DA (2016). The importance of surgical margins in pancreatic cancer. Journal of surgical oncology.

